# Supplementary material for: An open-source framework for large-scale, flexible evaluation of biomedical text mining systems
Source: J Biomed Discov Collab. 2008 Jan 29;3:1. doi: 10.1186/1747-5333-3-1 (PMC2276192; doi:10.1186/1747-5333-3-1)
Supplement: Additional file 1 — Evaluation platform source code. The additional file contains the software evaluation framework discussed in this paper. The most current release can be downloaded from SourceForge.net [1]. See the accompanying README file for instructions on installation and use. Also included in the distribution is a collection of UIMA wrappers for some commonly used BioNLP tools and annotated corpora. [file 1747-5333-3-1-S1.zip › bionlp_uima_repository-1.0.1/docs/api/src-html/edu/uchsc/ccp/uima/mention/CCPComplexSlotMention_Type.html]

```
001    
002    /* First created by JCasGen Tue Aug 28 16:44:10 MDT 2007 */
003    package edu.uchsc.ccp.uima.mention;
004    
005    import org.apache.uima.cas.Feature;
006    import org.apache.uima.cas.FeatureStructure;
007    import org.apache.uima.cas.Type;
008    import org.apache.uima.cas.impl.CASImpl;
009    import org.apache.uima.cas.impl.FSGenerator;
010    import org.apache.uima.cas.impl.FeatureImpl;
011    import org.apache.uima.cas.impl.TypeImpl;
012    import org.apache.uima.jcas.JCas;
013    import org.apache.uima.jcas.JCasRegistry;
014    
015    /** A slot mention is deemed "complex" when its slot filler is a class mention as opposed to a String (See non-complex slot mention for String fillers). An example of a complex slot mention is the "transported entity" slot for the protein-transport class which would be filled with a protein class mention.
016     * Updated by JCasGen Tue Aug 28 16:44:10 MDT 2007
017     * @generated */
018    public class CCPComplexSlotMention_Type extends CCPSlotMention_Type {
019      /** @generated */
020      protected FSGenerator getFSGenerator() {return fsGenerator;}
021      /** @generated */
022      private final FSGenerator fsGenerator = 
023        new FSGenerator() {
024          public FeatureStructure createFS(int addr, CASImpl cas) {
025                             if (CCPComplexSlotMention_Type.this.useExistingInstance) {
026                               // Return eq fs instance if already created
027                         FeatureStructure fs = CCPComplexSlotMention_Type.this.jcas.getJfsFromCaddr(addr);
028                         if (null == fs) {
029                           fs = new CCPComplexSlotMention(addr, CCPComplexSlotMention_Type.this);
030                               CCPComplexSlotMention_Type.this.jcas.putJfsFromCaddr(addr, fs);
031                               return fs;
032                         }
033                         return fs;
034            } else return new CCPComplexSlotMention(addr, CCPComplexSlotMention_Type.this);
035              }
036        };
037      /** @generated */
038      public final static int typeIndexID = CCPComplexSlotMention.typeIndexID;
039      /** @generated 
040         @modifiable */
041      public final static boolean featOkTst = JCasRegistry.getFeatOkTst("edu.uchsc.ccp.uima.mention.CCPComplexSlotMention");
042     
043      /** @generated */
044      final Feature casFeat_classMentions;
045      /** @generated */
046      final int     casFeatCode_classMentions;
047      /** @generated */ 
048      public int getClassMentions(int addr) {
049            if (featOkTst && casFeat_classMentions == null)
050          jcas.throwFeatMissing("classMentions", "edu.uchsc.ccp.uima.mention.CCPComplexSlotMention");
051        return ll_cas.ll_getRefValue(addr, casFeatCode_classMentions);
052      }
053      /** @generated */    
054      public void setClassMentions(int addr, int v) {
055            if (featOkTst && casFeat_classMentions == null)
056          jcas.throwFeatMissing("classMentions", "edu.uchsc.ccp.uima.mention.CCPComplexSlotMention");
057        ll_cas.ll_setRefValue(addr, casFeatCode_classMentions, v);}
058        
059       /** @generated */
060      public int getClassMentions(int addr, int i) {
061            if (featOkTst && casFeat_classMentions == null)
062          jcas.throwFeatMissing("classMentions", "edu.uchsc.ccp.uima.mention.CCPComplexSlotMention");
063        if (lowLevelTypeChecks)
064          return ll_cas.ll_getRefArrayValue(ll_cas.ll_getRefValue(addr, casFeatCode_classMentions), i, true);
065        jcas.checkArrayBounds(ll_cas.ll_getRefValue(addr, casFeatCode_classMentions), i);
066            return ll_cas.ll_getRefArrayValue(ll_cas.ll_getRefValue(addr, casFeatCode_classMentions), i);
067      }
068       
069      /** @generated */ 
070      public void setClassMentions(int addr, int i, int v) {
071            if (featOkTst && casFeat_classMentions == null)
072          jcas.throwFeatMissing("classMentions", "edu.uchsc.ccp.uima.mention.CCPComplexSlotMention");
073        if (lowLevelTypeChecks)
074          ll_cas.ll_setRefArrayValue(ll_cas.ll_getRefValue(addr, casFeatCode_classMentions), i, v, true);
075        jcas.checkArrayBounds(ll_cas.ll_getRefValue(addr, casFeatCode_classMentions), i);
076        ll_cas.ll_setRefArrayValue(ll_cas.ll_getRefValue(addr, casFeatCode_classMentions), i, v);
077      }
078     
079    
080    
081    
082      /** initialize variables to correspond with Cas Type and Features
083            * @generated */
084      public CCPComplexSlotMention_Type(JCas jcas, Type casType) {
085        super(jcas, casType);
086        casImpl.getFSClassRegistry().addGeneratorForType((TypeImpl)this.casType, getFSGenerator());
087    
088     
089        casFeat_classMentions = jcas.getRequiredFeatureDE(casType, "classMentions", "uima.cas.FSArray", featOkTst);
090        casFeatCode_classMentions  = (null == casFeat_classMentions) ? JCas.INVALID_FEATURE_CODE : ((FeatureImpl)casFeat_classMentions).getCode();
091    
092      }
093    }
094    
095    
096    
097
```
